# A Randomized Controlled Pilot Study of Topical Ropivacaine for Prevention of Post-POEM Pain

**DOI:** 10.1093/jcag/gwac007

**Published:** 2022-03-16

**Authors:** Mandip Rai, Amir Nazarin, Connie Taylor, Michael McMullen, Lawrence Hookey, Wiley Chung, Robert Bechara

**Affiliations:** Division of Gastroenterology, Queen’s University, Ontario, Canada; Department of Medicine, Queen’s University, Ontario, Canada; Division of Gastroenterology, Queen’s University, Ontario, Canada; Department of Anesthesiology and Perioperative Medicine, Queen’s University, Ontario, Canada; Division of Gastroenterology, Queen’s University, Ontario, Canada; Division of General Surgery and Thoracic Surgery, Queen’s University, Ontario, Canada; Division of Gastroenterology, Queen’s University, Ontario, Canada

**Keywords:** Achalasia, Dysphagia, POEM

## Abstract

**Background and Aims:**

Although usually mild to moderate in severity, postoperative pain after peroral endoscopic myotomy (POEM) is common. There are no studies that have addressed minimizing postoperative pain in patients undergoing POEM for achalasia. We hypothesized that intraoperative topical intra-tunnel irrigation with ropivacaine would result in a significant reduction in pain scores in the postoperative period.

**Methods:**

A double-blind, randomized, placebo-controlled trial was conducted at the Kingston Health Sciences Center. Patients received either 30 mL of 0.2% ropivacaine or 30 mL of placebo irrigated topically into the POEM tunnel after completing the myotomy and prior to closing the mucosal incision. The primary outcome was pain post-POEM at 6 h assessed by the Numeric Rating Scale (NRS). Secondary objectives included assessing pain score at 0.5, 1, 2, 4 h post-POEM and on discharge, Quality of Recovery (QoR-15) scores at discharge, narcotic requirement, adverse events, and patients’ willingness to have the procedure done on an outpatient basis.

**Results:**

A total of 20 patients were enrolled. For the primary outcome of pain post-POEM at 6 h, the NRS was 1.1 in the placebo group and 2.4 in the ropivacaine group (95% CI of the difference: −3.2 to 0.6, *P* = 0.171). No statistical difference was seen in the pain scores. Overall usage of post-procedural narcotics was low with no differences between the two groups. Fifty percent of patients in both groups were willing to have the procedure done as an outpatient.

**Conclusion:**

The addition of intra-procedural tunnel irrigation with 30 mL 0.2% ropivacaine did not lead to reduced post-POEM pain.

## INTRODUCTION/BACKGROUND

Achalasia is a disorder of esophageal motility characterized by a loss of enteric neurons resulting in impaired relaxation of the lower esophageal sphincter (LES) and absence of esophageal peristalsis (1). There are no current treatments that allow for regeneration of the enteric neurons. Interventions focus primarily on lowering the LES pressure to provide symptom relief and improve quality of life. Interventions include botulinum toxin injection, controlled pneumatic dilatation (PD), laparoscopic Heller myotomy (LHM) and, more recently, peroral endoscopic myotomy (POEM).

POEM has emerged as a minimally invasive treatment for achalasia and is the endoscopic equivalent to surgical myotomy. It is a first line treatment for achalasia and the preferred treatment for type 3 achalasia (2). A pooled analysis of the several randomized-controlled trials that compared LHM and POEM demonstrated similar efficacy, but higher postoperative complications and adverse events in LHM when compared with POEM (3). In addition, when compared with LHM, POEM has been shown to lead to significantly lower post-operative pain, opioid analgesic use and time to return to activities of daily living (4).

Although usually only mild to moderate in severity, postoperative pain after POEM is common. There are no studies that have addressed minimizing postoperative pain in patients undergoing POEM for achalasia. We currently use a multimodal approach to pain management with topical lidocaine, ketorolac, and liquid acetaminophen.

Ropivacaine is a commonly used local anesthetic in other minimally invasive surgeries. In a randomized controlled trial in patients undergoing laparoscopic cholecystectomy, shoulder pain which was assessed for up to 72 h post-operatively was significantly decreased in patients that received 10 mL of 0.25% bupivacaine instilled into the gallbladder bed versus patients that received 10 mL of normal saline (5). A systematic review and meta-analysis revealed that patients undergoing laparoscopic cholecystectomy that received intraperitoneal ropivacaine instillation had significantly lower pain scores as measured by the visual analogue scale (VAS) at 4–8 h and at 9–24 h and interestingly developed fewer adverse events when compared with control group (6).

We hypothesized that intraoperative topical intra-tunnel irrigation with ropivacaine would result in a significant reduction in pain scores and decreased requirements of additional analgesics in the postoperative period.

## METHODS

### Study Design and Setting

This was a randomized, double blind, placebo-controlled trial conducted at the Kingston Health Sciences Center (a tertiary care academic center) from June 2019 to December 2020. The operator and patients were blinded to the treatment. The research pharmacist was not blinded. Patients were randomized by an independent research pharmacist using blinded block randomization. All procedures were performed by a single operator (RB) as described previously (7).

### Inclusion Criteria

All patients 18 years of age and older undergoing POEM for achalasia who were able to provide informed consent were approached for participation. The diagnosis was based on high-resolution esophageal manometry (HRM) using the Chicago classification 3.0 when available (Sandhill, Milwaukee, WI) (8). If HRM was not available, the diagnosis was based on older manometric, endoscopic and radiologic information.

### Exclusion Criteria

Patients with known adverse reactions to local anesthetics and NSAIDs, (GFR < 50), chronic pain taking regular opioids (requiring daily opioid therapy > 30 mg morphine or equivalents), and patients unable to give informed consent were excluded.

### Study Intervention

The study solutions were prepared prior to the procedure by an independent research pharmacist and stored as per pharmacy standards. The study solutions consisted of either 30 mL of saline or 30 mL of 0.2% ropivacaine in 30 cc syringes. Both solutions were clear, colorless and could not be distinguished from one another based on appearance or consistency. On the day of the procedure, the study solution for each patient was obtained in a sealed envelope with anonymized randomization code. During the POEM procedure, after confirming the adequacy of the myotomy, the study solution was instilled into the tunnel through the working channel of the gastroscope. The mucosal incision was then sealed with hemostatic clips.

### Assessment of Pain

After the POEM procedure, pain was assessed by a blinded clinical research assistant at 0.5, 1, 2, 4 and 6 h as well as on discharge. The Numeric Rating Scale (NRS) and the Visual Descriptor Scale Score (VDS) are validated pain assessment tools that were used to assess and record pain scores (9).

1) **NRS:** The patient was asked to rate their pain on a scale of 0–10, 0 representing no pain, and 10 representing the worst pain they have ever felt in their life.
2) **VDS:** The patient was asked to indicate verbally the severity of their pain as one of the following: no pain, slight pain, mild pain, moderate pain, severe pain, extreme pain, most intense pain imaginable.

### Quality of Recovery (QoR-15) score

This validated measure captures the patient’s initial post-operative health condition and captures global assessment of patient’s recovery (10, 11). The patients were asked to rate various emotional and physical aspects of their post-operative condition as experienced in the past 24 h on a scale of 0–10. Zero representing an emotion or activity they experienced or accomplished none of time, and 10 representing an emotion or activity experienced or accomplished all of the time.

### Primary Outcome

1) Post-POEM pain at 6 h as assessed via the NRS.

### Secondary Outcomes

1) Post-POEM pain scores assessed by the NRS and Visual Descriptor Scale (VDS) at 0.5, 1, 2 and 4 h and on discharge (24–30 hours post-POEM)
2) Quality of Recovery (QoR-15) score (12) at discharge (24–30 h post-POEM)
3) Post-POEM opioid analgesic requirement
4) Adverse events
5) Patient’s willingness to have the procedure performed as an outpatient

### Anesthetic Technique

Anesthesia and post-operative care were protocolled for patients in this study. After intravenous induction and endotracheal intubation, anesthesia was maintained with sevoflurane and pain managed with fentanyl (25–50 mcg as needed, up to a maximum of 2 mcg/kg), as needed to minimize the effect on post-procedure pain. No long-acting opioids (morphine/hydromorphone) were administered. Immediately prior to extubating, all patients were given dexamethasone 6 mg intravenous (IV), ondansetron 4 mg IV and ketorolac 30 mg IV. The occurrence of post-POEM pain in the Post Anesthesia Care Unit (PACU) was managed with morphine 1–2 mg IV every 5 min as needed as per anesthesia standard practice. Post-POEM nausea and vomiting were treated with haloperidol 0.5 mg IV × 2 doses and dimenhydrinate 25 mg IV every 30 min × 2 doses as required. Post-POEM all patients were started on regular liquid acetaminophen 650mg orally every 4 h, 2% viscous lidocaine 15 mL orally four times per day, sucralfate suspension 1 g orally four times daily and ketorolac (as needed) 30 mg IV every 6 h.

### Sample Size and Statistics

This is a pilot study as there is a lack of studies to base the calculation of the sample size. A power calculation was performed for the primary outcome NRS score at 6 h post-POEM. A baseline NRS of 5 was assumed. We thought a clinically significant difference would be a reduction in NRS from 5 to 2. To detect a difference in the mean NRS score from 5 to 2, with SD estimated conservatively at ±2, 10 patients would be required in each group, with alpha set at 0.05 and power of 90%. Data are presented as frequencies and percentages, means (± standard deviation) or median (range) where appropriate. T-tests were used to compare the mean values for the two groups. All calculations were conducted in SPSS v23 (IBM, New York).

### Study Ethics and Registration

No industry-related funding was received to support this study or compensate study investigators. The study protocol was approved by the health sciences research ethics board at Queen’s University (DMED 2186-18). All patients were voluntarily consented during their clinic visit by a dedicated research coordinator. The study is registered on clinicaltrials.gov NCT03702647 (October 11, 2018).

## RESULTS

**Figure 1.**
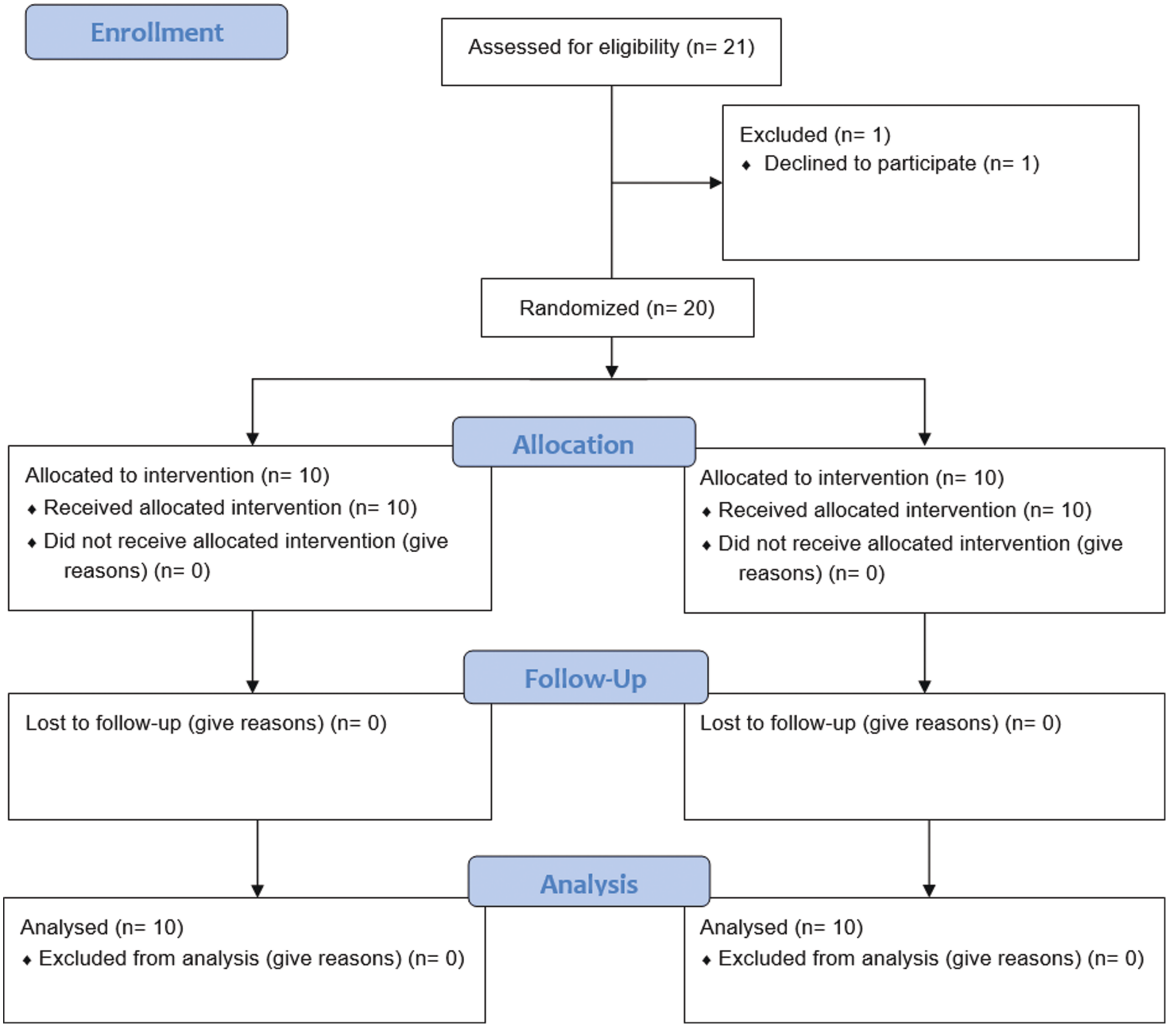
Consort flow diagram.

A total of 20 patients were enrolled, 10 in each group (Figure 1). Baseline patient characteristics are presented in Table 1. In the placebo group, 20% had type 1 achalasia, 30% type 2, 40% type 3 and 10% were unclassified. In the ropivacaine group, 60% had type 2 achalasia, 30% type 3 achalasia and 10% unclassified. The median symptoms duration was 121 months in the placebo group and 87 months in the ropivacaine group. Seventy percent of patients in the placebo group had prior treatment for achalasia compared to 60% in the ropivacaine group.

**Table 1.** Patient demographics

Characteristic	Placebo (*n* = 10)	Intervention (*n* = 10)
Male sex, no. (%)	6.0	8.0
Age, mean [SD]	58.0 [17.9]	52.2 [18.1]
BMI (kg/m^2^), mean [SD]	27.5 [3.0]	27.0 [7.2]
ASA class II	5.0	5.0
ASA class III	5.0	5.0
Achalasia Subtype, no. (%)		
Type 1	2.0 (20%)	0.0 (0%)
Type 2	3.0 (30%)	6.0 (60%)
Type 3	4.0 (40%)	3.0 (30%)
Unclassified	1.0 (10%)	1.0 (10%)
Integrated Relaxation Pressure (mmHg), median [IQR]	20.5 [16.0–31.0]	27.0 [19.5–33.0]
Symptom duration in months, median [IQR]	121.0 [81.0 242.50]	87.0 [51.0 - 135.0]
Patients with prior treatments, no. (%)	7.0 (70%)	6.0 (60%)
Pre-POEM Eckardt Score, median [IQR]	9.0 [7.8–13.3]	8.5 [8.0–11.0]

For the primary outcome of post-POEM pain at 6 h assessed by NRS, there was no statistically significant difference between placebo and ropivacaine groups, NRS 1.1 versus 2.4 (95% CI of the difference: −3.2 to 0.6, *P* = 0.171), respectively (Table 2). There was no significant difference in pain scores at 0.5, 1, 2, 4 h and on discharge post-POEM between placebo and ropivacaine groups. In addition, there was no significant difference between placebo and ropivacaine groups in the intra-procedural use of fentanyl, 87.5mcg (SD 46.0 mcg) versus 110.0 mcg (SD 62.6 mcg) (*P* = 0.372), respectively. No adverse events occurred with medications administration and there was no evidence of local anesthetic systemic toxicity. No adverse events occurred during or after the POEM procedure. No differences were seen in QoR-15 scores at discharge in either group (Table 3).

**Table 2.** Results of post-POEM pain scores

	Placebo (*n* = 10)	Ropivacaine (*n* = 10)	Mean Difference	95% CI of the Difference	*P*
NRS: 0.5 h, mean [SD]	5.3 [2.2]	4.2 [2.5]	1.1	−1.1 to 3.3	0.305
VDS: 0.5 h, mean [SD]	3.8 [1.1]	3.3 [1.4]	0.5	−0.7 to 1.7	0.396
NRS: 1.0 h, mean [SD]	4.5 [2.5]	3.8 [1.1]	0.7	−1.1 to 2.5	0.431
VDS: 1.0 h, mean [SD]	3.3 [1.1]	2.9 [0.9]	0.4	−0.5 to 1.3	0.370
NRS: 2.0 h, mean [SD]	2.9 [2.5]	3.5 [1.7]	−0.6	−2.6 to 1.4	0.533
VDS: 2.0 h, mean [SD]	2.5 [1.2]	2.7 [0.8]	−0.2	−1.2 to 0.8	0.665
NRS: 4.0 h, mean [SD]	1.6 [1.7]	2.3 [2.0]	−0.7	−2.4 to 1.0	0.406
VDS: 4.0 h, mean [SD]	1.8 [1.2]	2.1 [1.0]	−0.3	−1.4 to 0.8	0.556
NRS: 6.0 h, mean [SD]	1.1 [1.5]	2.4 [2.5]	−1.3	−3.2 to 0.6	0.171
VDS: 6.0 h, mean [SD]	1.6 [1.0]	2.2 [1.2]	−0.6	−1.6 to 0.4	0.241
NRS: Discharge, mean [SD]	0.9 [1.5]	1.3 [0.7]	−0.5	−1.6 to 0.7	0.406
VDS: Discharge, mean [SD]	1.5 [1.0]	1.7 [0.7]	−0.2	−1.0 to 0.6	0.600

**Table 3.** Quality of recovery (QoR-15) score

	Placebo (*n* = 10)	Ropivacaine (*n* = 10)	*P*
Q1–10. 0 = None, 10 = All the time			
Q1. Able to breath easily	9.3 [1.1]	9.1 [1.5]	0.729
Q2. Been able to enjoy food	3.2 [4.1]	2.3 [3.8]	0.616
Q3. Feeling rested	7.4 [3.1]	6.5 [2.6]	0.490
Q4. Have a good sleep	6.3 [3.0]	6.1 [3.0]	0.883
Q5. Able to look after personal toilet and hygiene unaided	9.7 [1.0]	9.7 [0.5]	1.00
Q6. Able to communicate with family and friends	9.8 [0.6]	9.3 [0.8]	0.145
Q7. Getting support from hospital doctors and nurses	9.5 [1.3]	9.4 [1.6]	0.878
Q8. Able to return to work or usual home activities	7.0 [4.3]	6.3 [4.1]	0.714
Q9. Feeling comfortable and in control	8.8 [2.0]	7.8 [3.4]	0.432
Q10. Having a feeling of general well-being	8.8 [1.6]	8.1 [2.6]	0.469
Q11–15. 0 = All of the time, 10 = None of the time			
Q11. Moderate pain	7.8 [3.1]	6.1 [3.0]	0.233
Q12. Severe pain	9.2 [2.2]	8.8 [2.1]	0.682
Q13. Nausea or vomiting	9.9 [0.3]	9.4 [1.1]	0.175
Q14. Feeling worried or anxious	7.8 [3.1]	8.6 [2.4]	0.520
Q15. Feeling sad or depressed	9.0 [2.5]	9.8 [0.6]	0.346

In both groups, the usage of opioids was low (30% Placebo group and 20% Ropivacaine group). Three out of 10 patients used a as needed dose in the placebo group and 2 out of 10 in the ropivacaine group. At 0.5-h post-POEM, one patient in the placebo group used 1 mg of morphine. At 1-h post-POEM, two patients in the placebo group used 1 mg of morphine each and one patient in the ropivacaine group used 1 mg of morphine. At 2 and 4 h post-POEM, one patient in each group used 1 mg of morphine. After 4 h post-POEM, there was no opioid requirement in either group. No patients required haloperidol or ketorolac. Fifty percent of patients in both groups were willing to have the procedure done as an outpatient.

## DISCUSSION

In this RCT, we found no significant difference between placebo and ropivacaine. At 0.5-h post-POEM and at discharge, the mean NRS was 5.3 (SD 2.2) and 0.9 (SD 1.5) in the placebo group versus 4.2 (SD 2.5) and 1.3 (SD 0.7) in the ropivacaine group with no statistical difference. These results are similar to prior published studies reporting post-POEM pain. In a 2017 review by Misra et al., the average first pain score was 4.6 (on a 0-10 numeric scale) in the PACU post-POEM and 3.3 once the patient was on the regular hospital floor (13). In a 2013 review of LHM versus POEM, post-procedure pain assessed by the VAS was 3.9 (+/−0.6) in the POEM group and 5.7 (+/−0.4) in the LHM group (*P* = 0.02) (4).

At our centre, a multimodal approach to post-POEM pain management with the use of topical viscous lidocaine, ketorolac, and liquid acetaminophen is used. In both groups, post-POEM requirement of opioids was low within the first 4 h, and no patients required opioids after 4 h. Pain scores steadily decreased over time in both groups as seen in Table 2. In our experience, when using this multimodal approach, narcotics are rarely needed post-POEM. On review of the results, it was noted that no patients required narcotics 4 h post-POEM.

Fifty percent of patients in both the placebo group and ropivacaine stated that they would be willing to have the procedure done as an outpatient. In a 2019 study looking at outcomes of 103 POEMs, 62.4% of patients were discharged safely on the same day (14). Their results suggest that in a select population, POEM can be safely performed as an outpatient procedure as complications are low in expert hands. Post-POEM pain was the primary reason for requiring admission and this highlights the importance of managing pain post-POEM. Thus, with the optimization of post-POEM pain control, select patients can have the procedure performed on outpatient basis without compromise in patient comfort or morbidity.

The strengths of this study include its design as investigators, patients and outcome assessors were blinded to the intervention. Despite no significant difference in pain scores between groups, we demonstrated that the use of a multimodal non-opioid regimen was adequate for all, with opioids rarely being needed. Limitations include this being a single centre, single operator study and thus generalizability is limited. There was a relatively small sample size, and there is a possibility that a small statistically significant difference between the groups was missed, although the clinical significance of such a difference would be negligible. Our baseline assumption for the primary outcome was a NRS of 5 but the results were much less than expected with a NRS of 1.1 in the placebo group. Therefore, our study would be underpowered to detect a difference. However, given the small difference in pain scores between the two groups, finding such a difference would not be clinically significant and we will not pursue a larger trial

In summary, the addition of intra-operative tunnel irrigation with 0.2% ropivacaine did not lead to reduced pain post-POEM. However, with the regular use of a multimodal post-POEM pain regimen with topical viscous lidocaine, IV ketorolac and liquid acetaminophen good pain control was achieved for all patients with minimal requirement of opioids.

## References

[1] Boeckxstaens GE, Zaninotto G, Richter JE. Achalasia. Lancet 2014;383:83–93. doi:10.1016/S0140-6736(13)60651-0. 23871090

[2] Kahrilas PJ, Katzka D, Richter JE. Clinical practice update: The use of per-oral endoscopic myotomy in achalasia: Expert review and best practice advice from the AGA Institute. Gastroenterology. 2017;153:1205–11. doi:10.1053/j.gastro.2017.10.001. 28989059 PMC5670013

[3] Schlottmann F, Luckett DJ, Fine J, Shaheen NJ, Patti MG. Laparoscopic heller myotomy versus peroral endoscopic myotomy (POEM) for achalasia: A systematic review and meta-analysis. Ann Surg. 2018;267:451–60. doi:10.1097/SLA.0000000000002311. 28549006

[4] Ujiki MB, Yetasook AK, Zapf M, Linn JG, Carbray JM, Denham W. Peroral endoscopic myotomy: A short-term comparison with the standard laparoscopic approach. Surgery. 2013;154(4):893–7; discussion 897; discussion 897-900. 10.1016/j.surg.2013.04.042. 24074429

[5] Gharaibeh KIA, Al-Jaberi TM. Bupivacaine instillation into gallbladder bed after laparoscopic cholecystectomy: Does it decrease shoulder pain? J Laparoendosc Adv Surg Tech. 2000;10:137–41. 10.1089/lap.2000.10.137 10883990

[6] Yong L, Guang B. Intraperitoneal ropivacaine instillation versus no intraperitoneal ropivacaine instillation for laparoscopic cholecystectomy: A systematic review and meta-analysis. Int J Surg. 2017;44:229–43. doi:10.1016/j.ijsu.2017.06.043. 28669869

[7] Rai M, Woo M, Bechara R. The Canadian POEM experience: The first 50 patients. J Can Assoc Gastroenterol. 2020;4(3):110–114. 34056528 10.1093/jcag/gwaa018 PMC8158644

[8] Kahrilas PJ, Bredenoord AJ, Fox M, et al. The Chicago classification of esophageal motility disorders, v3.0. Neurogastroenterol Motil. 2015;27(2):160–74. 10.1111/nmo.12477 PMC4308501 25469569

[9] Bendinger T, Plunkett N. Measurement in pain medicine. BJA Education 2016;16:310–5. doi:10.1093/bjaed/mkw014.

[10] Chazapis M, Walker EM, Rooms MA, Kamming D, Moonesinghe SR. Measuring quality of recovery-15 after day case surgery. Br J Anaesth. 2016;116:241–8. doi:10.1093/bja/aev413. 26787793

[11] Myles PS, Boney O, Botti M, et al. Systematic review and consensus definitions for the Standardised Endpoints in Perioperative Medicine (StEP) initiative: Patient comfort. Br J Anaesth. 2018;120:705–11. doi:10.1016/j.bja.2017.12.037. 29576111

[12] Stark PA, Myles PS, Burke JA. Development and psychometric evaluation of a postoperative quality of recovery score: The QoR-15. Anesthesiology. 2013;118:1332–40. doi:10.1097/ALN.0b013e318289b84b. 23411725

[13] Misra L, Fukami N, Nikolic K, Trentman TL. Peroral endoscopic myotomy: Procedural complications and pain management for the perioperative clinician. Med Devices (Auckl). 2017;10:53–9. doi:10.2147/MDER.S115632. 28260955 PMC5330187

[14] Benias PC, Korrapati P, Raphael KL, et al. Safety and feasibility of performing peroral endoscopic myotomy as an outpatient procedure with same-day discharge. Gastrointest Endos. 2019;90:570–8. 10.1016/j.gie.2019.04.247 31078571

